# Split Hemianterior Tibialis Turndown Muscle Flap for Coverage of Distal Leg Wounds With Preservation of Function

**Published:** 2014-03-21

**Authors:** Vinay Gundlapalli, John W. Gillespie, Chris D. Tzarnas

**Affiliations:** ^a^General Surgery Resident, Mercy Catholic Medical Center, Philadelphia, Pa; ^b^General Surgery Resident, Chistiana Hospital, Newark, Del; ^c^Chief of Plastic Surgery, Mercy Catholic Medical Center, Philadelphia, Pa

**Keywords:** muscle flap, tibialis anterior muscle, lower extremity wound, split muscle, tibialis anterior tendon

## Abstract

**Objective:** A hemisplit turndown tibialis anterior muscle flap is described for coverage of distal leg wounds with preservation of active extensor function for open wounds of the distal ankle is presented. This is a new flap not previously described and is another local option for coverage of selected distal leg wounds. **Methods:** A description of the operative procedure and a clinical successful example is presented. **Results:** The split hemitibialis anterior turndown muscle flap was successful and preserved function of the muscle and tendon. **Conclusions:** This is another option for coverage of difficult wounds of the lower extremity without sacrifice of function of the donor muscle.

Successful coverage of soft tissue defects in the distal third of the leg is one of the most challenging tasks for the reconstructive plastic surgeon. There are many options for the coverage of soft tissue defects in the distal third of the leg ranging from local flaps to microvascular free-tissue transfer.^1^^-^13 We describe a case of an open wound on the ankle, which was successfully treated with a novel local flap termed the split hemianterior tibialis turndown muscle flap. We believe this is another novel muscle flap not previously described for coverage of challenging wounds of the distal leg and ankle, while still maintaining function of the tibialis anterior tendon.

## METHODS

**Figure 1:**
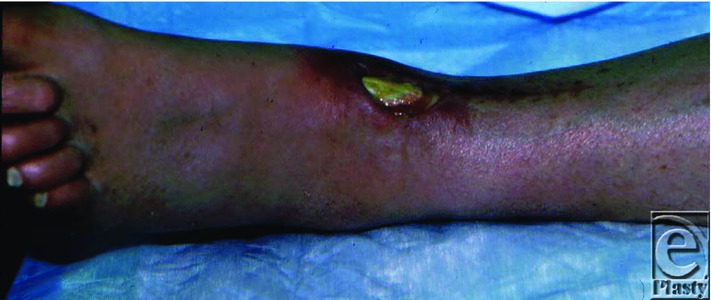
Left ankle wound with exposed tendon present for 3 months.

**Figure 2:**
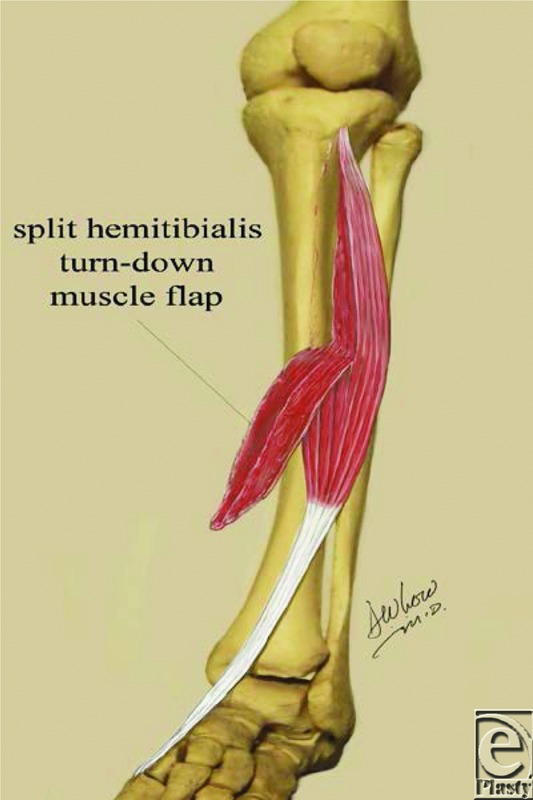
Cartoon of split hemitibialis anterior turn-down muscle flap demonstrating the intermuscular dissection using one-half of the muscle for coverage, preserving the remainder to maintain function of the muscle.

**Figure 3:**
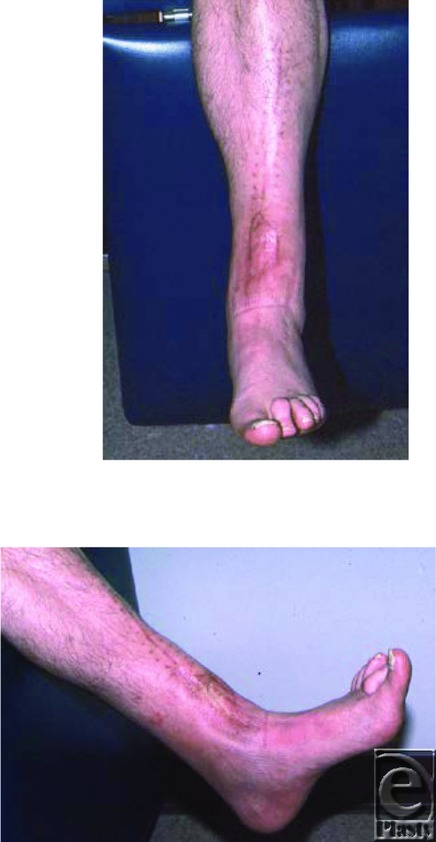
Result at 6 months postoperatively with full function and a closed stable wound.

A 53-year-old man sustained a severely comminuted fracture of the left ankle, from a crush injury at a construction site, which required open reduction and internal fixation with screws and plate. Subsequently, skin slough of the dorsal aspect of the ankle incision resulted in a 6 × 6 cm^2^ ulcer with exposed tibialis anterior tendon. Initially, the wound was managed conservatively with debridement and local wound care. This resulted in a 2 × 2 cm^2^ defect at the level of the ankle with exposure of the tendon of the anterior tibialis muscle that would not heal after several months of conservative wound care (Fig 1). He was referred to plastic surgery. On physical examination, there was a 2 × 2 cm^2^ full-thickness defect of the distal ankle with exposed tibialis anterior tendon. Distal pulses of the foot were intact and palpable. He underwent surgical treatment consisting of a split hemitibialis turndown muscle flap to cover the wound (Fig 2). The muscle flap was initially covered with a temporary xenograft to allow assessment of the viability of this muscle flap. Four days later, the turndown muscle flap appeared to be completely viable. There was no overt infection and the patient was returned to the operating room for removal of the xenograft and coverage of the viable tibialis anterior muscle flap with an autologous split-thickness skin graft. At the time of discharge, his wounds were healing well primarily and at 6 months of follow-up, the wound remained completely healed and function of the anterior tibialis tendon was intact as demonstrated by dorsiflexion of the foot and active extension of the great toe (Fig 3).

The split hemianterior tibialis turndown muscle flap was designed on the basis of the anatomic characteristics of its vascular supply, and the procedure is as follows.

With the patient supine on the operating room table, under spinal anesthesia, the left lower extremity was prepped and draped in a sterile fashion. A pneumatic tourniquet was applied. The dorsal incision was opened and carried cephalad along the anterior tibia, providing access to the anterior tibialis muscle. The wound was debrided. The lateral portion of the tibialis anterior muscle was split longitudinally, taking about one half of the muscle bulk commencing cephalad and coursing inferiorly and distally while preserving the tibialis anterior tendon. One-half of the muscle bulk was turned inferiorly on a series of distal intermuscular pedicles, by incising the muscle from cephalad to caudad, taking as few intermuscular perforators as possible to maintain blood flow and still permit turndown of the muscle flap to reach the defect. Care was taken not to completely separate the split muscle and to maintain a muscular connection with the remaining muscle bulk to permit preservation of the intermuscular vascular anatomy. Once the split turndown muscle flap reached the defect, the tourniquet was released. Bleeding points were clamped and tied. There was evidence of arterial vascular circulation in the muscle flap and the most distal portions of it appeared to have vascular circulation. The flap was inserted and the incision was closed. Xenograft dressing was placed over the muscle flap temporarily to enable flap viability assessment. Four days later, a split-thickness autologous skin graft was applied to the split turndown tibialis anterior muscle flap, which was completely viable and resulted in complete skin graft adherence to the muscle and graft survival with dorsiflexion function of the tibialis anterior tendon maintained.

## DISCUSSION

Wounds or defects in the lower leg are difficult to treat as the vital structures are covered with only skin and minimal soft tissue. The repair of these wounds either requires tissue with adequate blood supply or a microvascular free-tissue transfer. Over the last 3 decades, free flaps have been the treatment of choice for these hard to cover wounds. Transpositions of local muscles on a distal pedicle are not consistently reliable and alternatives include the cross-leg flap and the use of free flaps.

The choices of local flaps available for the distal third of the leg include the dorsalis pedis island,^4^ the extensor digitorum brevis muscle flap,^5^ the peroneal fascial flap,^6^ fasciocutaneous flaps based on the lateral calcaneal branch of peroneal artery^7^ or anterior perforating branch of peroneal artery,^8^ bipedal fasciocutaneous flap, distal flaps based on septocutaneous perforators of the posterior tibial^9^ or peroneal artery,^10^ and random fasciocutaneous flap.^11^ Propeller perforator flaps are a recent new option for small defects of the lower leg. However, they are associated with complications of total or partial flap loss from 5.5% to 11.6%.^12^ The choice and success of each of the aforementioned procedures depends on the anatomical and physiological variability of the individual patient. The degree of associated surrounding scarring and fibrosis often precludes safe use of some of the other local flap options, which occurred in this case and prompted the development of the split hemitibialis turndown muscle flap as an alternative flap. It also permitted us to avoid trying to develop a cutaneous or perforator flap surgery in a fibrotic and scarred region of the extremity resulting from the original traumatic crush injury. The flap described offers another alternative in selected cases.

The tibialis anterior partial muscle flap has been described to cover defects localized in the upper third and middle third of the tibial shaft, but not for wounds of the distal leg.^1^^,^^2^ Rotation of the superficial part of the muscle on a proximal pedicle while preserving its tendon and function can be done to cover defects in the upper third of the leg.

Several muscles have been split for coverage of difficult wounds. Robbins was the first to turn over the superficial part of the muscle as a fasciomuscle flap to cover an exposed tibia in the middle third of the leg, without causing a functional deficit.^2^ Subsequently, different variations of the longitudinal split technique have been reported with good results for coverage of compound tibial fracture,^12^ burns,^13^ and avulsion^1^ injuries of the middle third of the leg.

Splitting a muscle flap is not a new concept and has been described for other muscle flaps, which have been split including the latissimus dorsi and the pectoralis major muscle flap. The concept is based on the intermuscular vascular anatomy. The reach of the tibialis anterior muscle flap based on a proximal pedicle to cover the defects in the distal third of the leg is limited due to segmental short vascular pedicles. Extending the reach by the complete interruption of the musculotendinous unit leaves a considerable functional deficit and therefore is not recommended in ambulatory patients.^14^

The tibialis anterior muscle originates from the lateral condyle, upper half of the lateral surface of tibia, interosseous membrane, and crural fascia. The tendinous structure runs in the center of the muscle, becomes cordlike at the lower part, and inserts onto the first metatarsal and medial cuneiform bone, and it is responsible for dorsiflexion and inversion of the foot.^1^ The blood supply for the muscles originates from the anterior tibialis artery through 8 to 12 short segmental vessels.^1^^,^^2^ The distal cluster is most often found 4 to 9 cm proximal to the intermalleolar line.^3^ The concept is that intermuscular vascular connections will maintain circulation to the split portion of the muscle that is used as a turndown flap.

## RESULTS

An ankle soft tissue defect with exposed tendon and bone was covered by splitting the tibialis anterior muscle longitudinally and turning down the split muscle flap on a distal intermuscular pedicle, while preserving its function. This turndown flap technique, to our knowledge, has not been previously described but is based on sound anatomical concepts. The split hemitibialis anterior turndown muscle flap was successful in covering an ankle wound with exposed tendon and bone and preserved active dorsiflexion of the ankle and active extension of the great toe despite using a portion of the muscle for wound coverage. The wound coverage remained stable at 6 months follow-up with active ambulation and no wound healing issues. Active dorsiflexion of the foot and active extension of the great toe were present.

## CONCLUSION

The split hemitibialis anterior turndown muscle flap, for distal lower leg wounds, adds another local procedure to the armamentarium of a reconstructive plastic surgeon, a simple but effective local flap procedure to manage these difficult soft tissue defects in selected patients with adequate vascular supply of the limb.
